# Accuracy and efficiency of respiratory gating comparable to deep inspiration breath hold for pancreatic cancer treatment

**DOI:** 10.1002/acm2.13137

**Published:** 2020-12-30

**Authors:** Chuan Zeng, Xiang Li, Wei Lu, Marsha Reyngold, Richard M. Gewanter, John J. Cuaron, Ellen Yorke, Tianfang Li

**Affiliations:** ^1^ Department of Medical Physics Memorial Sloan Kettering Cancer Center New York NY USA; ^2^ Department of Radiation Oncology Memorial Sloan Kettering Cancer Center New York NY USA

**Keywords:** deep inspiration breath hold, intrafraction motion, pancreatic cancer, radiation therapy, respiratory gating

## Abstract

**Purpose:**

Deep inspiration breath hold (DIBH) and respiratory gating (RG) are widely used to reduce movement of target and healthy organs caused by breathing during irradiation. We hypothesized that accuracy and efficiency comparable to DIBH can be achieved with RG for pancreas treatment.

**Methods and Materials:**

Twenty consecutive patients with pancreatic cancer treated with DIBH (eight) or RG (twelve) volumetric modulated arc therapy during 2017–2019 were included in this study, with radiopaque markers implanted near or in the targets. Seventeen patients received 25 fractions, while the other three received 15 fractions. Only patients who could not tolerate DIBH received RG treatment. While both techniques relied on respiratory signals from external markers, internal target motions were monitored with kV X‐ray imaging during treatment. A 3‐mm external gating window was used for DIBH treatment; RG treatment was centered on end‐expiration with a duty cycle of 40%, corresponding to an external gating window of 2–3 mm. During dose delivery, kV images were automatically taken every 20^◦^ or 40^◦^ gantry rotation, from which internal markers were identified. The marker displacement from their initial positions and the residual motion amplitudes were calculated. For the analysis of treatment efficiency, the treatment time of every session was calculated from the motion management waveform files recorded at the treatment console.

**Results:**

Within one fraction, the displacement was 0–5 mm for DIBH and 0–6 mm for RG. The average magnitude of displacement for each patient during the entire course of treatment ranged 0–3 mm for both techniques. No statistically significant difference in displacement or residual motion was observed between the two techniques. The average treatment time was 15 min for DIBH and 17 min for RG, with no statistical significance.

**Conclusions:**

The accuracy and efficiency were comparable between RG and DIBH treatment for pancreas irradiation. RG is a feasible alternative strategy to DIBH.

## 1 INTRODUCTION

For locally advanced unresectable pancreatic cancer, conventional dose radiotherapy cannot effectively improve long‐term survival, while stereotactic body radiotherapy (SBRT) or hypofractionated ablative radiotherapy (in 15–25 fractions) has shown promising local control and an acceptable rate of adverse events.^1^, ^2^, ^3^ For these types of treatment, proper organ motion management is essential to avoid severe complications for three reasons: First, the pancreas is in proximity to a few critical structures such as the duodenum, stomach, kidneys, and spinal cord.^4^ Second, the pancreas undergoes significant respiration‐induced motion,^5^ which may lead to underdosage in parts of the tumor and overdosage to the organs at risk (OAR). Third, the target doses are significantly exceeding the tolerance of the surrounding normal tissues.

There have been many efforts to characterize pancreatic tumor movement during free breathing.^5^, ^6^, ^7^, ^8^, ^9^ Four‐dimensional computed tomography (4DCT) is a technique widely available for respiratory motion assessment. However, it has been shown to be inadequate in predicting intrafractional tumor motion.^10^, ^11^, ^12^, ^13^, ^14^, ^15^ Gierga *et* *al*. observed the motion of fiducial clips using fluoroscopy and found that the range of average motion in the superior‐inferior (SI) direction was 4–12 mm, and the motion in the anterior‐posterior (AP) direction was much smaller.^7^ CyberKnife^®^ (Accuray Incorporated, Sunnyvale, CA, USA) analysis showed mean respiratory motion ranged 0–9 mm and 1–5 mm, in the SI and left‐right directions, respectively.^16^ Feng *et* *al*. reported, based on cine MRI (magnetic resonance imaging) studies, that the tumor border movement was much larger than normal expectation.^8^ They reported that, although variable, the magnitude of pancreatic tumor movement might be as much as 4 cm in the SI direction. Movement in the AP direction was small (0–1 cm). The lateral movement was negligible.

A few strategies have been developed in radiotherapy to manage respiration‐induced tumor and organ motions, ranging from passive (such as internal margin expansion) to active (such as abdominal compression, breath hold, gating, or dynamic tumor tracking) approaches.^17^, ^18^, ^19^, ^20^, ^21^, ^22^, ^23^ Each technique provides different trade‐offs between ease of use, patient comfort, and the extent of motion reduction. In this study, we used two different approaches to limit the breathing motion during the treatment of pancreatic cancer — deep inspiration breath hold (DIBH) and respiratory gating (RG). Both strategies have been used for the treatment of breast cancer, lung cancer, and liver cancer.^24^, ^25^, ^26^, ^27^, ^28^, ^29^ Typically, both techniques rely on some external surrogate to reflect internal target movement, which may be a one‐dimensional (1D) signal or three‐dimensional (3D) surface information. Several groups have studied the correlation between external surrogate motion and internal target motion in breast cancer patients.^26^, ^28^ Dawson *et al*. studied the reproducibility of organ movement under active breathing control in eight patients with liver cancer by repeating fluoroscopy *before* each treatment.^24^ Very recently, we tracked the internal targets using kV images triggered *during* each DIBH treatment throughout the treatment course.^30^

DIBH requires patient cooperation and additional staff time and effort.^25^ The need for active patient participation and compliance severely limits the number of patients who can benefit from it. In contrast, with normal breathing, the requirements for RG are lower and make it more generally available for patients. Either technique may fit the needs of an individual patient. To the best of our knowledge, the effectiveness of tumor motion management and efficiency of DIBH and RG on pancreatic cancer treatment have not been compared. In this work, we used on‐treatment kV X‐ray imaging to monitor the internal tumor motion for DIBH and RG to characterize their efficacy for pancreatic cancer treatments in order to better inform between the two techniques and guide the clinical decision between the two. Internal targets, represented by radiopaque markers, were tracked using the kV X‐ray images triggered during treatment delivery.^23^, ^29^, ^30^, ^31^ We characterized the residual motion as the maximum observed motion amplitude in each fraction and analyzed the displacement vectors from the initial setup position to the positions observed in the triggered images. The efficiency of treatment delivery in terms of treatment time was also compared by the recorded acquisition time of the motion management waveform files from all treatment sessions.

## 2 METHODS

### 2.1 Patients

An institutional review board / privacy board data exemption was approved before the study. The study included twelve consecutive patients with pancreatic cancer who were treated with RG and eight with DIBH between 2017 and 2019, and each patient had some radiopaque markers (fiducial markers, surgical clips from attempted resection, and/or biliary stents, *etc*.) implanted near or inside the target. Table 1 shows patient demographic and treatment characteristics. Seventeen patients received 25 fractions for 75 Gy total dose to gross tumor volume (GTV) and 45 Gy to microscopic disease; the other three patients received 67.5 Gy and 37.5 Gy in 15 fractions.^3^ (An ongoing phase II clinical trial (NCT03523312) is evaluating these doses in a prospective manner.) Volumetric modulated arc therapy (VMAT) technique was used for treatment on TrueBeam linear accelerators (Varian Medical Systems, Palo Alto, CA, USA), which can acquire kV images during MV beam delivery using the gantry‐mounted kV imager (On‐Board Imager, Varian Medical Systems).^23^ Only patients who could not tolerate DIBH received RG treatment.

**Table 1:** Patient demographic and treatment characteristics.

Parameter	DIBH (8)	RG (12)
Age/year		
Median (range)	66 (48–91)^a^	76 (59–86)^a^
Gender		
Male	4 (50%)	5 (42%)
Female	4 (50%)	7 (58%)
Number of fractions		
Median (range)	25 (25–25)	25 (15–25)

Abbreviations: DIBH, deep inspiration breath hold; RG, respiratory gating.

^a^ *P* < 0.05 (two‐sample *t*‐test).

### 2.2 CT simulation

CT simulation scans were performed by physicians and therapists using the Brilliance Big Bore CT simulators (Philips Medical Systems, Cleveland, OH, USA). Patients were immobilized in customized body‐conformal molds (Alpha Cradle; Smithers Medical Products, North Canton, OH, USA) in supine position with arms raised.

The Varian Real‐time Positioning Management (RPM) system (Varian Medical Systems) was used to monitor patients’ breathing with the plastic block placed on abdomen. No compression device was applied. Patients were selected based on their ability to follow breath hold instructions and reproducibility of their breath hold pattern. Intravenous contrast was administered before performing the DIBH CT scan, and a second DIBH scan was usually performed at a later contrast phase. One of the DIBH scans was used to plan treatment based on target visibility. Patients who could not tolerate DIBH underwent 4DCT simulation and were to be treated with RG. Four‐dimensional CT data were binned into ten phases, with end‐inhale denoted as 0%. A free‐breathing CT scan was also taken as a backup. The patient respiratory trace recorded in RPM was imported into the treatment delivery system to be used as a reference for treatment.

### 2.3 Treatment planning

The CT datasets were transferred to the treatment planning system Eclipse (Varian Medical Systems). The attending physician outlined the GTV including tumors and metastatic lymph nodes in the patients’ DIBH (for DIBH treatment) or end‐exhale (for RG treatment) CT data. No other special treatment planning measures were taken for RG patients. The planning target volume (PTV) was created with a PTV margin of 3–5 mm and edited as needed for normal tissue protection.^2^, ^3^ The margin depended on the amount of contact and proximity to the OARs. High‐dose PTV was generated by expanding the GTV by 5 mm. Then the expanded OARs were subtracted from it. We do not utilize an internal target volume (ITV) approach for either DIBH or RG. There was no tumor motion in the DIBH scan, and tumor motion was confirmed to be <5 mm between 30% phase and 70% phase for RG patients (corresponding to end of exhalation with 40% duty cycle), obviating the need for ITV in either case. The fiducial markers were identified during the planning process for later comparison. VMAT treatment plans were developed, the planning goals of which were to meet the normal tissue constraints of the department (Table 2), while covering the PTV according to the prescription (low‐dose PTV *V*_100%_≥95%, high‐dose PTV *V*_100%_≥90% and *D*_max_ ≤ 110%). Our gastrointestinal (GI) OAR constraints are equivalent to reported constraints for conventional fractionation. The bowel dose constraints are based on a previous analysis.^32^ For most cases, all planning goals could be achieved with two or three arcs; however, more arcs were added if necessary.

**Table 2:** Normal tissue constraints.

		15 fractions	25 fractions
Small bowel	*D*max	40 Gy	55 Gy
Stomach	*D*max	45 Gy	60 Gy
Duodenum	*D*max	45 Gy	60 Gy
Large bowel	*D*max	50 Gy	65 Gy
Liver (non‐GTV)	*D*_max_ (if total volume *V* ≤ 700 cc)	24 Gy	28 Gy
	*D_V_* − _700 cc_ (if total volume *V* > 700 cc)	24 Gy	28 Gy
	*D*mean	24 Gy	28 Gy
Kidneys	*V*20 Gy	50%	
Single functioning kidney	*V*20 Gy	33%	

Abbreviation: GTV, gross tumor volume.

The attending physician can decide whether to exceed one or more of these constraints.

### 2.4 Treatment delivery and intrafraction imaging

All treatments were delivered on TrueBeam linear accelerators (Varian Medical Systems) with RPM systems. Before each treatment, the RPM block was placed at the same position as tattooed in the simulation (on abdomen inferior of the xiphoid process). Visual Coaching Device (Varian Medical System) was not available for this patient cohort.

For DIBH treatment, the patient received instructions to maintain DIBH, while cone beam CT (CBCT) was performed in a stop‐and‐go acquisition mode to obtain a complete volumetric dataset under breath hold. Then, the CBCT images were aligned to the planning DIBH CT matching on the implanted fiducial markers, and the couch was shifted accordingly. During the treatment, the patient was instructed to perform DIBH so that the RPM signal was voluntarily maintained in the 3‐mm gating window.

**Fig. 1:**
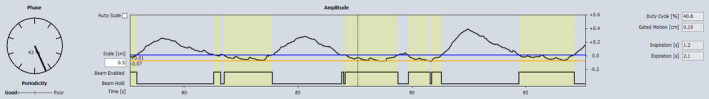
The user interface displaying the gating window on the patient’s Respiratory Waveform and thresholds (orange and blue horizontal lines) set for amplitude‐based gating at expiration. The square wave graph at the bottom of the display indicates when the treatment beam was enabled. The Measured Values display and the Periodicity Meter are also shown.

For RG treatment, CBCT was taken in the gating window and registered to the planning end‐exhale CT matching on the fiducial markers. The couch was shifted accordingly. RG treatment was centered at the end of exhalation with a duty cycle of 40%, which corresponded to a 2–3‐mm window (Fig. 1). Amplitude gating was used for all patients.^25^ CBCT acquisition started when the breathing curve entered the gating window (30% phase). Since CBCT acquisition took more than several seconds, it spanned over the several phases within the gating window (30–70%).

For both DIBH and RG treatments, if the patient’s breath was not within the gating window during treatment, the MV beam would be held automatically until the RPM trace returned to the gating window. A time delay of 0.5 s was applied to DIBH to allow the breath to reach the middle of the gating window. The acquisition of kV images was automatically triggered every 20^◦^ or 40^◦^ gantry rotation (since VMAT plans were used) through the Intrafraction Motion Review (IMR) application (Varian Medical Systems) while the MV beam was on. Since gantry rotation was uncorrelated with breathing motion, kV image captures were expected to be distributed randomly among the gated breathing phases. Each acquired kV image was displayed with graphically superimposed fiducial contours to help the therapists evaluate the intrafraction movement. Two‐millimeter expansions of the fiducial markers were also provided for reference to help the motion evaluation. Under monitoring using IMR, if there were large offsets (>3 mm) on two consecutive kV images, the treating therapists would intervene by repeating setup imaging. All kV images collected during each gantry rotation were combined into a movie and automatically saved to the image review system. For a typical 3‐arc fraction, approximately 27 images would be acquired, resulting in a total of more than 600 images for the entire treatment course of each patient.^30^ Triggered images outside of the gating window were excluded from our study.

### 2.5 Motion analysis

In order to track changes in the target position during treatment, we first needed to identify fiducial markers in or near the target in the setup CBCT images and record their 3D room coordinates so that we could compare them with the corresponding fiducial positions found in the triggered images. According to CBCT measurements, the length of a typical fiducial marker was about 6 mm. The number of fiducial markers varied from patient to patient. If multiple fiducial markers were available, one was selected and analyzed for each patient based on discernibility and relevance to the target. If a stent was specified as the matching structure for image guidance, it would also be used in this study.

Specifically, the triggered images were retrospectively reviewed in the Offline Review Workspace (Varian Medical Systems). Each kV image was examined carefully; and the same fiducial marker was identified in each frame. During the VMAT delivery, as the gantry rotated, the fiducial marker position changed in the kV images. The pixel positions of both ends of the fiducial marker in the two‐dimensional (2D) images were manually recorded and the center position calculated from the average of both ends.

To account for the geometric amplification of X‐ray projection, the pixel information was then converted into room coordinates based on the source‐to‐detector geometry. Basically, the fiducial marker in the kV image was back‐projected onto the plane where the marker was in the CBCT image. For a single 2D kV image, we can only reliably detect motion in the SI direction (and the direction tangent to kV imager rotation). However, since SI motion is usually the largest in the three directions, as other researchers have reported in the study of free‐breathing pancreatic tumor motion,^6^, ^7^, ^8^, ^9^, ^29^ we believe it is sufficient to characterize pancreatic tumor movement with only the SI coordinates of the fiducial markers. In this back‐projection calculation, marker coordinates in the AP and lateral directions determined from the CBCT contributed to the magnification factors from their physical locations to the imaging plane. During treatment, movement along the AP or lateral direction may affect the magnification factor and introduce an error proportional to the ratio of movement amplitude to the source‐axis distance (SAD). Since the motion along the AP or the lateral direction was much smaller than the kV SAD of 100 cm, in our estimation of SI motion, this error of less than 1% is negligible. For a more detailed description, see Ref. [30].

### 2.6 Treatment time

For the analysis of treatment efficiency, all the motion management waveform files recorded at the treatment console for every session were parsed programmatically. The treatment time for each session was calculated as the time from acquiring the first waveform file to the end of the last waveform file, that is, the time from the first setup imaging to the end of the last treatment beam. This general definition of treatment time includes the time taken to image the patient and adjust the setup afterwards, all pauses to adjust the gating thresholds, relocalize the target, and the actual delivery of the treatment beams. It is thus an indicator of the efficiency of treatment room usage.

### 2.7 Statistical methods

Two‐sample *t*‐test was used to compare patient age, number of fractions, motion characteristics, and treatment time between DIBH and RG cohorts. Patient gender was compared via the Wilcoxon rank sum test.

## 3 RESULTS

The RG cohort was significantly older than the DIBH cohort (*P* = 0.02; Table 1). No significant difference was observed in gender (*P* = 0.8) or number of fractions between the two cohorts.

**Fig. 2:**
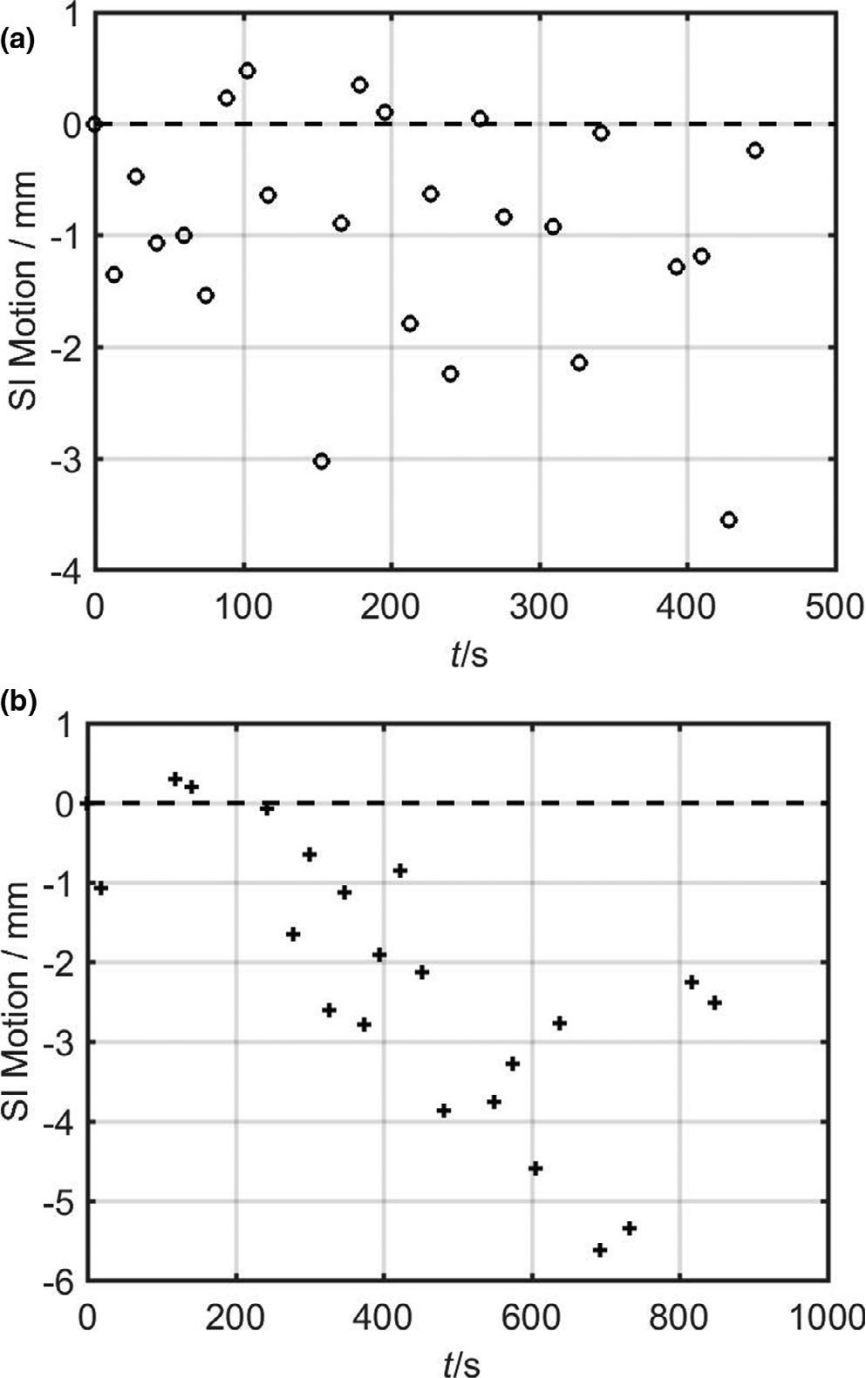
The superior‐inferior (SI) motion of a fiducial marker for one patient during two different respiratory‐gated treatment sessions. In one session the motion was random (a); in the other session, drift was evident, and treatment pause to relocate the target was necessary and thus longer treatment time (b).

Figure 2 shows an example of changes in the position of a patient’s fiducial marker during two different fractions of RG treatment. It was found that for this patient, the maximum residual motion under RG was about 8 mm throughout the treatment course (which happened in a fraction not shown in Fig. 2).

The motion characteristics, namely, the residual motion and the displacements, of all patients are listed in Tables 3 and 4 and summarized in Table 5 to compare the effectiveness of the two techniques of motion management. The residual motion study reveals the internal motion range of the patient when receiving DIBH or RG treatments. The displacement is the instantaneous distance from the setup position (in which target was aligned) during the treatment, which is directly related to the beam targeting accuracy. Table 5 also shows the statistics of the displacement amplitude of all patients. The average position of six of eight DIBH patients and seven of twelve RG patients was within 2 mm. The average magnitude of the overall displacement for the population was 1 mm, and the standard deviation (SD) was 1–2 mm.

**Table 3:** Residual motion and average displacement over the whole course of all deep inspiration breath hold patients. Positive displacement indicates the superior direction.

Patient number	Residual motion/mm	Average displacement/mm
1	5	0
2	8	−3
3	5	0
4	8	2
5	6	−1
6	5	0
7	7	2
8	3	−1

**Table 4:** Residual motion and average displacement over the whole course of all respiratory gating patients. Positive displacement indicates the superior direction.

Patient number	Residual motion/mm	Average displacement/mm
1	7	0
2	7	0
3	8	−2
4	6	3
5	10	2
6	6	3
7	4	0
8	6	−1
9	4	3
10	5	0
11	7	0
12	6	0

**Table 5:** Comparison of motion characteristics and treatment time of all patients.

Parameter	DIBH	RG
Residual motion over the whole course / mm Mean (range)	6 (3–8)^a^	6 (4–10)^a^
Mean magnitude of displacement^∗^ in one fraction / mm Range	0–5^a^	0–6^a^
Mean magnitude of displacement^∗^ over the whole course / mm Range	0–3^a^	0–3^a^
Treatment time / min Mean ± SD	15 ± 3^a^	17 ± 4^a^

Abbreviations: DIBH, deep inspiration breath hold; RG, respiratory gating.

^a^ *P* > 0.05 (two‐sample *t*‐test).

Evidently, the internal residual motion could be greater than the external RPM marker motion for some patients on some particular days. For four‐in‐eight DIBH patients and three‐in‐twelve RG patients, the average SI internal motion was within 5 mm. Instantaneous residual motion of as large as 1 cm was observed, but this offset may not persist throughout the session.

The treatment time, including imaging time, was in average 15 min for DIBH and 17 min for RG, with 3–4 min SD (Table 5).

There was no significant difference in residual motion (*P* = 0.4), displacement amplitude (*P* = 0.9), or treatment time (*P* = 0.3) between the DIBH cohort and the RG cohort.

## 4 DISCUSSION

**Fig. 3:**
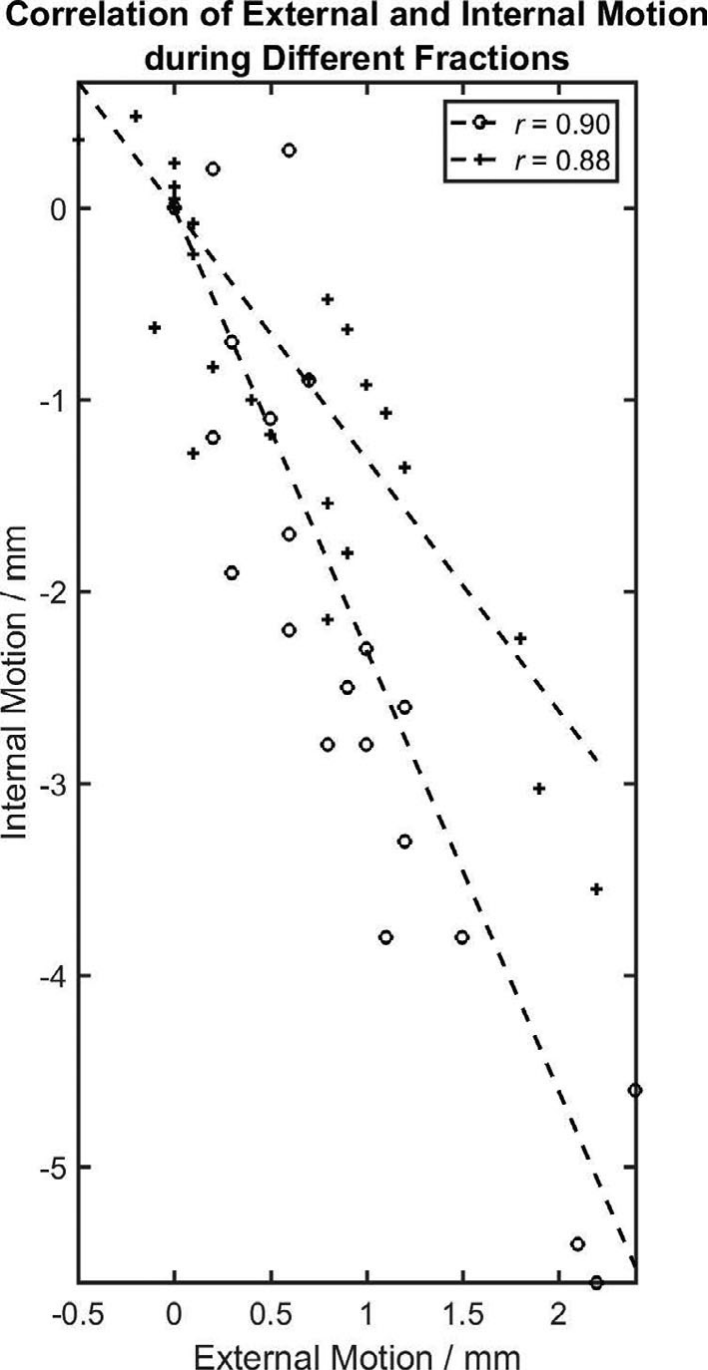
The correlation of the superior‐inferior motion of a fiducial marker to the external marker motion for one patient during two different respiratory‐gated treatment sessions (circle ‘◦’ and plus sign ‘+’). The dependence was highly linear in both fractions, as shown by the least‐square linear fit (dashed lines). However, the dependence varied quantitatively by about a factor of two in slope.

In this study, we evaluated and compared the internal target movements using real‐time kV imaging during DIBH and RG treatments of pancreatic cancer. Although both techniques limited the movement of the external RPM marker to about 3 mm, the amplitudes of the internal motions were usually larger, averaging about 6 mm, and in some extreme cases 1 cm. The accuracy of position determination from kV images was limited by the imager pixel size, which corresponds to ~0.3 mm at the isocenter. The origin of the inaccuracy was attributed to the in‐equivalency of internal motion and external motion. Our result is consistent with other studies on the correlation between external surrogate movements and internal tumor movements.^26^, ^27^, ^28^, ^33^, ^34^ In Fig. 3, we plotted the internal motion as a function of the external signal (extracted from the RPM Respiratory Waveforms) for the two sessions in Fig. 2. The dependence was highly linear in both fractions, with correlation coefficients ∼0.9. However, quantitatively, the slope of the dependence varied by about a factor of two for the two different fractions. This daily change in the correlation between internal movements and external signals may be unpredictable, which highlights the need for real‐time monitoring of internal targets.

As mentioned in Ref. [30], for a typical patient, *D*_95%_ of the high‐dose PTV dropped by about 5% with 3‐mm SI offset; >10% with 5‐mm offset; and >20% with 1‐cm offset. Ding *et al*. established a range of motion limits for safe delivery of pancreatic SBRT.^35^ For 33 Gy delivered in five fractions, a conservative 6.3‐mm limit was considered safe for all 91 patients in their study. That limit would drop to 4.2 mm if the dose was to be escalated to 50 Gy. Vinogradskiy *et al*. evaluated the dosimetric impact of positional corrections made according to kV real‐time monitoring in pancreatic SBRT.^29^ They concluded that with a 5‐mm PTV margin, 55% of observed corrections resulted in no noticeable target dose difference, while the rest 45% of corrections resulted in an average point dose difference of 23% ± 22% of the prescribed dose. That is, if real‐time monitoring was not available and positional corrections were not made, the delivered dose may be >20% different from the planned dose.

When the average displacement for each patient was applied to the treatment plan as an isocenter shift, the GTV coverage (*V*_100%_) changed by <1% for both DIBH and RG groups, with 4% and 2% SDs for DIBH and RG, respectively.

While we have demonstrated comparable residual motion for RG patients and DIBH patients, a significant underestimate could result from the simulation 4DCT for the RG patients. The residual motion observed in the simulation 4DCT within the gating window (end‐exhale ± 20% phase) was on average 3 mm (range 2–6 mm), which was significantly smaller than the actual residual motion during treatment (*P* = 10^−4^; paired‐sample *t*‐test). That difference also confirmed the inadequacy of a single 4DCT in representing respiration‐induced motion magnitude of pancreatic tumors over a 3–5‐week radiotherapy scheme.^11^, ^15^

Our RG patient group was about ten years older than the DIBH group (Table 1), which may be intuitively linked to our selection criterion for RG (only patients who could not tolerate DIBH were treated with RG). Despite the significant difference in age and duty cycle (40% for RG versus 100% for DIBH), comparable treatment efficiency was achieved for the RG group relative to the DIBH group. This implies that the time of coaching the patient may be significant for DIBH in every fraction. On the other hand, if the two groups of patients were of comparable age, the treatment efficiency for RG may be even higher than DIBH, which warrants future investigation.

We currently use amplitude gating for all patients because earlier versions of the RPM software were more robust in amplitude than phase gating.^25^ For the RG cohort in this study, phase gating was evaluated retrospectively, and the robustness was still inferior to amplitude gating, the details of which will be published in a subsequent report.

One of the limitations of this study is the lack of real‐time location information of OARs, because kV‐visible markers were only present near or inside the targets. Our assumption is that during VMAT delivery, the target is monitored in real‐time through different angles, and the surrounding organs are unlikely to move significantly and systematically into the treatment field. Moreover, the recent introduction of MR‐guided radiotherapy offers the possibility to monitor OARs during treatment using a high‐temporal cine MRI,^36^ from which the position of healthy organs could be quantified.

While we found that the treatment times were not statistically different between DIBH and RG, the average difference of 2 min might have clinical significance.

The main focus of our paper is to use X‐ray imaging to *directly* monitor internal target motion during DIBH and RG treatments and compare the efficacy and efficiency of the two motion management techniques. These treatments typically rely on some sort of surrogate, which may be a 1D RPM signal or 3D surface guidance. We applied our method to the RPM‐based treatments in this work, since this is the current practice at our institution. However, an on‐treatment X‐ray imaging method can also be applied to surface‐guided treatments. We emphasize here that X‐ray imaging provides true information about internal target location for patients undergoing DIBH and RG treatments.

Rong *et al*. concluded that the RPM system alone might not be sufficient for left‐sided breast / chest wall DIBH treatments, and Vision RT / Align RT, a 3D surface guidance technique, was superior.^26^ Fassi *et al*. also reported that 3D optical monitoring of multiple surface control points might help to optimize the use of RPM system for left breast DIBH irradiation and increase the robustness of external surrogates for DIBH guidance. According to a study surveying 530 radiation oncologists in the United States,^37^ RPM is the most commonly used DIBH system (54%), with the second being Vision RT/Align RT (31%). Furthermore, published studies on surface tracking are mostly for breast cancer.^26^, ^27^, ^28^, ^37^ The conclusion drawn from our study may or may not be applicable to 3D surface tracking. However, even if 3D surface guidance was utilized, one would still need a means of monitoring internal target motion especially for GI targets. Our real‐time kV imaging can be applied along with surface guidance as well.

## 5 CONCLUSION

The accuracy and efficiency of RG treatment was comparable to DIBH in pancreas irradiation. RG is a viable alternative strategy to DIBH. Our result will benefit a large portion of the radiation oncology society that uses the RPM system. The clinical outcome of a larger cohort study on ablative treatment will be presented in a subsequent report.

## Conflicts of Interest

The author have no other relevant conflict of interest to disclose.

## Author Contributions

Dr. Zeng had full access to all of the data in the study and takes responsibility of the integrity of the data and the accuracy of the data analysis. Concept and design: Zeng, X. Li, T. Li. Acquisition, analysis, or interpretation of data: Zeng, X. Li, Lu, Reyngold, Gewanter, Cuaron, Yorke, T. Li. Drafting of the manuscript: Zeng, T. Li. Critical revision of the manuscript for important intellectual content: Zeng, Lu, Gewanter, Yorke, T. Li. Statistical analysis: Zeng. Obtaining funding: X. Li, T. Li. Administrative, technical, or material support: Zeng, T. Li. Supervision: Zeng, X. Li, T. Li.
